# Pulmonary Tuberculosis among Male Inmates in the Largest Prison of Eastern Nepal

**DOI:** 10.1155/2019/3176167

**Published:** 2019-10-09

**Authors:** Gambhir Shrestha, Deepak Kumar Yadav, Rabin Gautam, Rashmi Mulmi, Dharanidhar Baral, Paras Kumar Pokharel

**Affiliations:** ^1^Department of Cancer Prevention, Control and Research, B.P. Koirala Memorial Cancer Hospital, Bharatpur, Chitwan, Nepal; ^2^School of Public Health and Community Medicine, B.P. Koirala Institute of Health Sciences, Dharan, Sunsari, Nepal; ^3^National Tuberculosis Program, Health Research and Social Development Forum, Thapathali, Kathmandu, Nepal

## Abstract

**Introduction:**

The prevalence of Pulmonary Tuberculosis (PTB) is much higher in the prison population than in the general population. This study aims to find out the prevalence of PTB and its associated factors among inmates in eastern Nepal.

**Methods:**

This cross-sectional study was conducted in Jhumka Regional Prison, the largest male prison of Eastern Nepal from September 2014 to August 2015. Semi-structured questionnaires were used to identify individuals with a cough more than one-week duration among 434 randomly selected inmates. Screening of PTB was done by sputum smear test and/or GeneXpert test. Prevalence of TB was defined as the number of cases detected during the study period divided by the total number of inmates screened during that period. Fisher's exact test was used to find out the association of PTB with related variables.

**Results:**

A total of 434 inmates were screened for PTB with mean age 35.7 years and body mass index 22.7 kg/m^2^. A total of 68 inmates had a productive cough of more than a week and two patients were already on anti-tuberculosis therapy at the time of screening. Sixty sputum samples were considered for sputum smear/GeneXpert test and 6 of them (10%) had positive results. The prevalence of TB in the Prison was 1843/100,000 population. Chest pain and abnormal chest auscultation findings were found to be significantly associated with PTB.

**Conclusions:**

This study showed that there was a high rate of PTB among inmates in Nepal. The results suggest a need for effective screening of PTB and strategies to improve management including reduction of PTB transmission in the prison.

## 1. Background

The prevalence of infectious diseases, chronic diseases, blood-borne viruses, and sexually transmitted diseases are much higher in the prison population than in the general population [1–7]. Pulmonary Tuberculosis (PTB) has been reported up to 100 times more in the prison population than the civilian population [8]. First, higher rates of TB in the prison setting has been attributed to prisoners who are from population groups already at high risk of TB infection and disease (addicted to alcohol, drug users, homelessness, living in unfavorable social conditions such as poverty and misery). Second, prisons promote transmission of TB infection through prolonged and repeated exposure due to overcrowding, inadequate ventilation, and late case detection. Third, poor nutritional status and coexisting pathology like HIV in prison predispose them to higher chances of TB transmission [9].

TB is a serious cause of morbidity and mortality in prison among the inmates. The prison system often faces various problems that hinder TB control lack of laboratory facility and other diagnostic tools, interrupted supply and stock out of drugs, inadequate infection control measures, lack of coordination between civilian and prison health services, and low priority for health care to inmates [10].

TB remains a major public health problem in Nepal and is the sixth leading cause of death in the country. With a population of around 30 million, the number of cases registered at National TB Program as of 2015 includes 34121 cases (case notification rate 123/100,000) with approximately 8000–10,000 cases to be missing every year. As of 2015, the estimated prevalence rate was 211/100,000 population, and with the annual estimated incidence of 158/100000 [11, 12].

Prison is one of the areas where Urban TB control program (DOTS) has been implemented in Nepal; however, there is inadequate human resource along with the absence of a key focal person to look after TB in prisons. Furthermore, there are no surveillance data available regarding tuberculosis in prison setting in Nepal. To our best knowledge, this is the first study of its kind to find out the prevalence and associated factors of PTB in a Nepalese prison.

## 2. Methods

### 2.1. Study Setting and Design

Screening of PTB among inmates was conducted in Jhumka Regional Prison, the largest adult male prison in the Eastern Development Region of Nepal from September 2014 to August 2015. The prison capacity is of 1500 prisoners.

### 2.2. Sampling Technique

This paper is a part of a large study and has followed the methods of Shrestha et al. 2017 [13]. The health needs, risky behaviours, and depression among the inmates of same population have been presented elsewhere [13, 14]. Briefly, out of total 1203 inmates in the prison, 450 were randomly selected, of whom 434 gave consent for the study.

### 2.3. Ethical Approval

Ethical Review Board approval was obtained from B.P. Koirala Institute of Health Sciences. The Department of Prison Management, Ministry of Home Affairs, Government of Nepal granted permission to conduct study in the prison. Informed written consent from the participants was taken prior to the interview.

### 2.4. Methods of Data Collection

All eligible participants were interviewed in-person by a community physician, who collected detailed information on socio-demographic characteristics, imprisonment characteristics, substance use disorders, and symptoms of tuberculosis using a semi-structured questionnaire. A detailed clinical examination was also performed.

### 2.5. Screening for Tuberculosis

**Figure 1:**
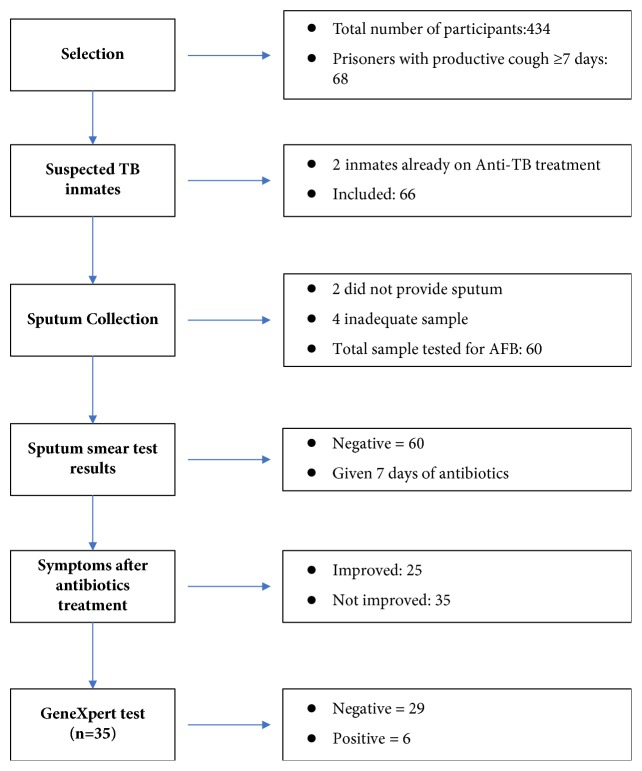
Screening of PTB among inmates in the prison.

Prisoners with a productive cough more than a week were considered TB suspects and two sputum samples (one on-the-spot and second early morning samples) were collected for Ziehl–Neelsen (ZN) sputum smear test. The acid-fast bacilli microscopy was performed by a certified laboratory technician in nearby Baklori Health Post following the guidelines set by the National TB Program (NTP). The NTP guideline recommends use of nonTB antibiotics and restricts use of quinolone group of drugs in chest symptomatic individuals [15]. In this study, patients with negative smear test were treated with 7 days' course of antibiotics (Amoxicillin) as per the NTP guidelines. Those whose symptoms did not improve were considered for GeneXpert test at B.P. Koirala Institute of Health Sciences for suspected sputum smears (Figure 1). All the inmates with PTB were placed on anti-tuberculosis treatment. Prevalence of TB in prison was calculated as the number of cases diagnosed during the study period divided by the total number of inmates screened during that period multiplied by 100,000 and expressed as cases per 100,000.

### 2.6. Nutritional Status

The nutritional status of the inmates was classified according to body mass index into (i) underweight with BMI less than 18.5 kg/m^2^ (ii) normal with range 18.5–24.99 kg/m^2^ (iii) pre-obese 25–29.99 kg/m^2^, obese class I 30–34.99 kg/m^2^ and severe obesity ≥40 kg/m^2^.

### 2.7. Statistical Analysis

In this study, the dependent variable was TB among inmates and the independent variables were sociodemographic characteristics, incarceration profile, substance abuse, and symptoms of TB. Only one inmate in the study population was known HIV seropositive, and hence we did not look for its association with TB. Descriptive statistics were used to present the dependent and independent variables. Chi-square test (Fisher's exact test where applicable) was performed to determine the association of tuberculosis with the independent variables. Statistical Package for Social Sciences (SPSS, version 20) was used for all statistical analyses. A *p*-value of <0.05 was considered as statistically significant.

## 3. Results

The age of the participants ranged from 18 to 81 years with a mean of 35.73 years (SD 13.25). About 172 (39.6%) of the participating inmates had a cough at the time of interview and 79 (45.9%) of them had the productive type of cough, and among these with productive cough, 68 (86.1%) had a cough of 7 days and more. About 42.2% of the inmates reported recent loss of appetite and more than half (55.1%) reported loss of weight during the last 3 months. Very few (1.8%) had a fever at the time of interview and 9.2% reported of having chest pain. Inmates with a past history of tuberculosis were 5.1% (Table 1).

The mean BMI was found to be 22.76 (SD 2.93) which ranged from 15 to 33 kg/m^2^. About three-fourths of the inmates were found to have normal range of BMI while only 6% were underweight, 19% were pre-obese and 2% were Obese class I (Table 2).

A total of 68 inmates had a productive cough ≥7 days of which two were already on anti-tuberculosis therapy and hence 66 inmates were suspected of TB. Two inmates did not provide a sputum sample so 64 inmates were considered for sputum smear/GeneXpert test for tuberculosis. Four samples were rejected due to inadequate sample and hence 60 samples were tested. All the sample were negative for sputum AFB smear test. After 7 days of antibiotics treatment, 25 inmates symptomatically improved. Remaining 35 inmates did not improve and one sample was sent for GeneXpert test. Six samples (10%) had a positive test result for TB in GeneXpert test of which one had rifampicin resistant (Figure 1). Of the 434 inmates evaluated, 6 newly diagnosed and 2 under anti-tubercular treatment i.e., 8 inmates (1.84%) had active tuberculosis in the prison i.e., the prevalence of TB in Jhumka Regional Prison was **1843/100,000** population.

Age, education, duration of stay in prison, previous incarceration, overcrowding, and substance use disorders were not found to statistically significant with the prevalence of tuberculosis (Table 3).

In this study, fever, duration of cough, weight loss, loss of appetite, BMI, and past history of TB were not found to be the significantly associated with tuberculosis. Only chest pain and abnormal chest finding on auscultation (i.e., wheezes and crackles) were found to be statistically significant with the presence of tuberculosis (*p* = 0.012 and 0.027, respectively) (Table 4).

## 4. Discussion

The prevalence of PTB in Nepalese prison was found to be 1843/100000 population, which is 8.7 times higher than the general population. This study reflects important insights into the burden of tuberculosis in the country, particularly in the high-risk prison setting. The detection of 6 new undiagnosed PTB cases shows the vulnerability of the jail inmates for the disease. The prevalence of tuberculosis is similar to other studies [16–18]. However, the rate of tuberculosis in prisoners compared with that in the general population varies depending on the prison location [19]. The high prevalence of PTB in prison settings highlights the importance of implementing systematic tuberculosis screening program in correctional facilities within the country. Such high prevalence could have a potential impact on the overall national TB control program due to potential spread to and through visitors, prison staffs, and discharged inmates themselves [18].

In this study, there was no significant association between the age group with the disease. This finding is in accordance with other studies [18, 20]. It was also observed that more older age group populations (>45 years) have TB compared to the younger people. This is in contrast to the study done in Africa, where the primary age group affected has been the younger age group [17, 21].

In this study, the duration of cough did not have any association with tuberculosis. This is similar to the study done by Moges et al. [18]. Although, fever and cough are cardinal symptoms for diagnosis of tuberculosis and various studies have pointed out an association of these symptoms with TB disease [16, 17, 22]. Our lack of association could be due to the substantial lag time associated between symptoms and diagnosis. However, even in this study, we saw the association of inmates with history of chest pain and tuberculosis similar to other studies [17, 23]. Abnormal chest finding during auscultation was found to be a associated with PTB. Along with the symptoms, a careful and detailed clinical examination can help in PTB diagnosis. This association has not been tested in other prison studies, hence further research can be done in this regard.

Weight loss, loss of appetite, and malnutrition have been important risk factors associated with tuberculosis in various studies [18, 20, 24]. However, this study does not show this association. Some other studies also do not show this association [16, 17].

The present study also did not find an association between substance use disorder, overcrowding, length of incarceration and previous incarceration. Various studies conducted have similar findings [18, 21, 25, 26]. In contrast to this, a study conducted in Cameroon found a positive association of prison stay of less than 12 months and a history of previous incarceration with PTB [27]. This observation could be explained by the low number of confirmed TB cases detected to confer any association with these factors with tuberculosis.

This study highlights considerable strength and weaknesses. To our best knowledge, this is the first study which shows the high burden of Tuberculosis in a prison setting in Nepal. Prison health is sadly neglected and under-addressed in most parts of the world [28]. Nepal in line with many countries does not have specific prison health policies. Our study findings underline the importance of periodic and timely screening and diagnosis of TB in prisons and timely institution of TB treatment to control the transmission of the infection [29]. Proper screening for tuberculosis symptoms is highly recommended as the first line of defence in prison.

We also acknowledge the limitation of our study where we could do screening among only 434 inmates (small sample size) due to the control prison setting environment. Furthermore, our diagnosis mostly relied on the sputum microscopy examination, where the yield is substantially lower compared to the modern diagnostic methods like GeneXpert. Not all sputum samples could be sent for GeneXpert due to resource constraint of GeneXpert machines in setting like Nepal. Sputum culture, and X-ray screening of suspected inmates were not done, which could have further helped diagnose TB.

Furthermore, the cross-sectional design of this study does not allow us to make definitive inferences about the effect of the risk factors in association with tuberculosis. Lastly, as this study is done only in one male prison, it cannot be generalized to other prisons of the country.

## 5. Conclusions

This study found a high prevalence of PTB among inmates in Nepal than the general population. Our study highlights the vulnerability of inmates to tuberculosis and stresses the need for effective screening for TB in new and continuing prisoners and appropriate measures to halt the transmission of the disease in the high-risk prison environment. Isolation of active PTB patients and early diagnosis and treatment should be implemented to control the disease and to reduce the incidence of TB in prisons.

## Figures and Tables

**Table 1:** Symptoms of tuberculosis among inmates in the prison (*n* = 434).

Symptoms of TB	*n* (%)
Respiratory symptoms	

*Cough*	
Yes	172 (39.6)
No	262 (60.4)

*Type of cough* (*n* = 172)	
Productive	79 (45.9)
Nonproductive	93 (54.1)

*Duration of productive cough in days* (*n* = 79)	
<7	11 (13.9)
≥7	68 (86.1)

*Chest pain*	
Yes	40 (9.2)
No	394 (90.8)

General symptoms	

*Loss of appetite*	
Yes	181 (42.2)
No	251 (57.8)

*Weight loss in 3 months*	
Yes	239 (55.1)
No	195 (44.9)

*Fever*	
Yes	8 (1.8)
No	426 (98.2)

*Past history of TB*	
Yes	22 (5.1)
No	412 (94.9)

**Table 2:** Nutritional status of inmates in the prison (*n* = 434).

Nutritional status (kg/m^2^)	*n* (%)
Underweight (<18.5)	25 (5.8)
Normal (18.5–24.99)	317 (73.0)
Pre‐obese (25–29.99)	84 (19.4)
Obese Class I (30–34.99)	8 (1.8)

**Table 3:** Association of TB among inmates with related variables.

Characteristics	Total suspected TB (*n* = 68)	TB suspects with sputum test (*n* = 60)	Inmates with TB (*n* = 6)	Inmates without TB (*n* = 54)	*p*‐value
*Age (in years)*					0.179
18–44	42 (61.8)	38 (63.3)	2 (5.3)	36 (94.7)	
≥45	26 (38.2)	22 (36.7)	4 (18.2)	18 (81.8)	

*Education*					0.223
Illiterate	28 (41.2)	25 (41.7)	4 (16.0)	21 (84.0)	
Literate	40 (58.8)	35 (58.3)	2 (5.7)	33 (94.3)	

*Time held in prison*					0.541
<12 months	7 (10.3)	7 (11.7)	1 (14.3)	6 (85.7)	
≥12 months	61 (89.7)	53 (88.3)	5 (9.4)	48 (90.6)	

*Previous incarceration*					0.052
Yes	13 (19.1)	10 (16.7)	3 (30.0)	7 (70.0)	
No	55 (80.9)	50 (83.3)	3 (6.0)	47 (94.0)	

*Inmates per cell*					1.000
≤50	36 (52.9)	31 (51.7)	3 (9.7)	28 (90.3)	
>50	32 (47.1)	29 (48.3)	3 (10.3)	26 (89.7)	

*Alcohol intake*					1.000
Yes	48 (70.6)	43 (71.7)	4 (9.3)	39 (90.7)	
Never	20 (29.4)	17 (28.3)	2 (11.8)	15 (88.2)	

*Smoking status*					0.675
Current user	36 (52.9)	32 (53.3)	4 (12.5)	28 (87.5)	
Nonuser/Ex‐user	32 (47.1)	28 (46.7)	2 (7.1)	26 (92.9)	

*Chewing tobacco*					0.681
Current user	37 (54.4)	33 (55.0)	4 (12.1)	29 (87.9)	
Nonuser/Ex‐user	31 (45.6)	27 (45.0)	2 (7.4)	25 (92.6)	

*Illicit drug use*					0.581
Yes	15 (22.1)	11 (18.3)	0 (0.0)	11 (100)	
No	53 (77.9)	49 (81.7)	6 (12.2)	43 (87.8)	

*Injectable drug use*					0.275
Yes	6 (8.8)	3 (5.0)	1 (33.3)	2 (66.7)	
No	62 (91.2)	57 (95.0)	5 (8.8)	52 (91.2)	

**Table 4:** Association of TB among inmates with signs and symptoms.

Signs/Symptoms	Total suspected TB (*n* = 68)	TB suspects with sputum test (*n* = 60)	Inmates with TB (*n* = 6)	Inmates without TB (*n* = 54)	*p*-value
*Fever*					0.484
Yes	6 (8.8)	6 (10.0)	1 (16.7)	5 (83.3)	
No	62 (91.2)	54 (90.0)	5 (9.3)	49 (90.7)	

*Duration of cough*					0.370
≤4 weeks	47 (69.1)	41 (68.3)	3 (7.3)	38 (92.7)	
>4 weeks	21 (30.9)	19 (19.7)	3 (15.8)	16 (84.2)	

*Weight loss in 3 months*					0.658
Yes	47 (69.1)	42 (70.0)	5 (11.9)	37 (88.1)	
No	21 (30.9)	18 (30.0)	1 (5.6)	17 (94.4)	

*Recent loss of appetite*					0.671
Yes	37 (54.4)	30 (50.0)	4 (13.3)	26 (86.7)	
No	31 (45.6)	30 (50.0)	2 (6.7)	28 (93.3)	

*Chest pain*					0.012^*^
Yes	13 (19.1)	12 (20.0)	4 (33.3)	8 (66.7)	
No	55 (80.9)	48 (80.0)	2 (4.2)	46 (95.8)	

*Chest auscultation^#^*					0.027^*^
Abnormal	8 (88.2)	8 (13.3)	3 (37.5)	5 (62.5)	
Normal	60 (11.8)	52 (86.7)	3 (5.8)	49 (94.2)	

*BMI (kg/m^2^)*					0.421
<18.5	7 (10.3)	5 (8.3)	1 (20)	4 (80)	
≥18.5	61 (89.7)	55 (91.7)	5 (9.1)	50 (90.9)	

*Past history of TB*					0.484
Yes	6 (8.8)	6 (10.0)	1 (16.7)	5 (83.3)	
No	62 (91.2)	54 (90.0)	5 (9.3)	49 (90.7)	

^*^Significant/ ^#^only wheezes and crackles were found on auscultation.

## Data Availability

The datasets used and/or analyzed during the current study are available from the corresponding author upon reasonable request.

## Abbreviations

TB: Tuberculosis
PTB: Pulmonary tuberculosis
HIV: Human immunodeficiency virus
BMI: Body mass index
NTP: National tuberculosis program
ZN: Ziehl–Neelsen
ATT: Anti-tubercular treatment.
